# Percentile-Based Normative Standards for Strength Assessment Beyond Linear Bodyweight Multipliers in Competitive Powerlifters

**DOI:** 10.1186/s40798-026-01111-z

**Published:** 2026-09-16

**Authors:** Simone Montenegro, Pamela Wicker, Tim Wiedenmann, Ludwig Rappelt, Lars Donath

**Affiliations:** 1 Department of Applied Exercise Science, German Sport University Cologne, Cologne, Germany; 2 Department of Medicine and Aging Sciences, Università degli Studi “G. d’Annunzio” Chieti-Pescara, Chieti, Italy; 3 Department of Neurosciences, Biomedicine and Movement, University of Verona, Verona, Italy; 4 Department of Sports Science, Bielefeld University, Bielefeld, Germany

**Keywords:** Normative standards, Bodyweight multipliers, Powerlifting, Strength training

## Abstract

**Background:**

Bodyweight multipliers (BM = L/M, where L denotes the absolute load lifted in kilograms and M the athlete's recorded body mass) represent the dominant benchmarking heuristic in strength and conditioning. However, because relative strength declines non-linearly with body mass, a single fixed threshold is not equally attainable across the body-mass spectrum, and these static standards provide no lift-specific, percentile-based reference for practitioner use.

**Methods:**

A large-scale dataset of drug-tested, classic competitive powerlifters was analyzed to establish empirical percentile-based normative standards. A "Best-Ever" filter was applied to isolate each athlete's peak performance, yielding a final sample of *N* = 101,898 athletes (aged 21–49). Quantile regression was employed to independently estimate normative percentiles (50th, 75th, 90th, and 99th) and evaluate mass-dependent changes in relative strength across all three lifts. A Loess smoothing procedure was applied to percentile values summarized across weight classes to visualize mass-dependent performance trends. All athletes were retrospectively mapped into current International Powerlifting Federation (IPF) weight categories to produce absolute-load normative atlases stratified by lift, sex, and weight class. The internal lift ratio, the percentage contribution of each lift to the competitive total, was additionally quantified across the mass spectrum.

**Results:**

A systematic, non-linear decrease in relative BMs was observed across all performance percentiles (*p* < 0.001). The smoothed male 99th percentile deadlift multiplier declined from 4.37 in the 59 kg class to 2.65 in the 120+ kg category. A progressive mass-dependent decline was identified, with the rate of relative strength decay becoming significantly steeper at elite tiers (99th percentile) compared to intermediate levels (50th percentile). Female athletes exhibited a steeper mass-dependent decline in bench press and squat performance compared to males. The internal lift ratio shifted systematically from deadlift-dominance in lightweight categories (~ 42%–45%) to a substantially narrower gap between deadlift and squat contribution in heavyweight classes (~ 39% vs ~37%).

**Conclusion:**

Traditional linear BM benchmarks are not equally attainable across body-mass classes and lack the resolution to support lift-specific athlete assessment. The empirical percentile-based normative framework established in this study provides coaches and researchers with a transparent, data-driven tool that accurately reflects the distribution of competitive performance across body mass categories. A practical normative atlas expressed in absolute kilograms is provided as a directly applicable field tool.

**Supplementary Information:**

The online version contains supplementary material available at https://doi.org/10.1186/s40798-026-01111-z.

## Key Points

- Traditional linear bodyweight multipliers are not equally attainable across the body-mass spectrum: relative strength declines systematically and non-linearly with increasing body mass, meaning that heavier athletes are structurally constrained from achieving the same standards applied to lighter competitors.
- The mass-dependent decline in relative strength intensifies at elite performance levels, with world-class athletes facing a disproportionately steeper decay compared to intermediate lifters, a finding with direct implications for weight class transition strategies.
- Percentile-based normative atlases stratified by sex, IPF weight class, and individual lift provide coaches and practitioners with a transparent, calculation-free tool for lift-specific athlete assessment and evidence-based weight class transition planning.

**Supplementary Information:**

The online version contains supplementary material available at https://doi.org/10.1186/s40798-026-01111-z.

## Introduction

The quantification of muscular strength relative to body mass represents a foundational pillar of athletic assessment, particularly within disciplines where performance is stratified by weight categories [1, 2]. Powerlifting, a strength sport contested through the maximal back squat, bench press, and deadlift, distinct from weightlifting and strongman, is stratified by sex and body-mass category, making it a uniquely tractable model for studying the body mass–strength relationship. The same three lifts also serve as foundational testing and training exercises throughout strength and conditioning (S&C), so benchmarks derived from competitive powerlifting carry relevance beyond the sport itself. In the practical environment of powerlifting and S&C, coaches and practitioners have historically relied upon Bodyweight Multipliers (BM = L/M, where L denotes the absolute load lifted in kilograms and M the athlete's recorded body mass) as an intuitive benchmarking heuristic [3]. While the International Powerlifting Federation Good Lift (IPF GL) points serve as the official method for cross-weight class comparison and are formally embedded via the rulebook, linear bodyweight multipliers such as the "double-bodyweight squat" or the "triple-bodyweight deadlift" remain pervasive informal heuristics in the general strength and conditioning culture, serving as markers of athletic status and training readiness [3, 4].

Despite their intuitive appeal and ease of application, these static linear benchmarks rest on a fundamental physiological assumption that does not hold across the full body mass spectrum: that strength scales in a direct, 1-to-1 ratio with total body mass. This assumption is inconsistent with well-established biological scaling principles. As body mass increases, muscle cross-sectional area, the primary driver of force production, scales at a lower rate than total body volume and mass, meaning that heavier athletes are structurally constrained to achieve lower strength-to-mass ratios than their lighter counterparts [6, 7]. As a practical consequence, a benchmark such as the "2.5× bodyweight deadlift" is readily attainable for a 60 kg athlete but approaches the absolute frontier of achievable competitive performance for a 130 kg athlete, so that the same numerical standard represents a markedly different level of difficulty depending on the athlete's body mass. The difficulty, however, lies not in the relative-strength ratio itself, a legitimate descriptor of how strength differs across body-mass classes, but in the application of a single fixed threshold across the full spectrum. These multipliers are, moreover, informal training references and do not determine competitive outcomes, which are decided within weight classes and, across classes, by dedicated ranking coefficients; the limitation is thus one of benchmarking resolution, not of competition scoring.

Previous attempts to address this imbalance have taken two directions, neither of which fully serves the needs of practitioners. Allometric scaling approaches apply a corrective exponent to body mass to produce a more biologically grounded relative strength score [7–9]. While theoretically sound, these methods yield abstract numerical scores that are difficult to interpret in a coaching context and do not translate into lift-specific, actionable targets. Competitive ranking coefficients such as the Wilks formula [10, 11], the IPF GL formula, the DOTS coefficient, and the Sinclair coefficient in weightlifting [12], and their more recent iterations, are optimized to identify the best lifter across weight classes. The IPF GL formula, for instance, is re-derived periodically by regression on a reference sample of elite totals, and its comparative performance against alternative formulations has been formally evaluated by the International Powerlifting Federation itself [13]. However, because of this specialized scope, aggregate coefficients are not intended to serve as day-to-day assessment tools. A coefficient score does not tell a coach whether an athlete's squat is disproportionately weak compared to the rest of the competitive total, nor does it provide an absolute kilogram target for a specific lift. Furthermore, aggregate coefficients treat overall performance as a single entity, which inherently limits their ability to account for how the internal balance between the squat, bench press, and deadlift shifts systematically as an athlete's body mass increases (although it is worth noting that the IPF GL formula provides a separate calculation for bench-press-only events) [14–16]. In practice, knowing whether an 83 kg athlete's 180 kg squat places them at the 50th or the 90th percentile of drug-tested competitors worldwide, and whether their deadlift or bench press sits at a comparable percentile, requires normative data of this kind; an allometric score alone cannot answer this.

That strength scales non-linearly with body mass is itself well established [6, 7, 17]. Translating this relationship into practical benchmarks, however, requires empirical normative data, and contemporary percentiles for competitive powerlifting have recently been reported, most comprehensively by van den Hoek et al. [4, 18], who analyzed 809,986 competition entries and documented their secular evolution, with the associated Global Strength Norms Calculator by The Strength Initiative (www.thestrengthinitiative.com) [4] providing the corresponding practitioner-facing descriptive tool. What remains underdeveloped is a framework that retains one peak performance per unique athlete within a homogeneous, contemporary sample; models the mass-dependent decline itself, testing whether its rate steepens across performance tiers; characterizes how the internal contribution of the three lifts shifts with body mass; and translates the norms into lift-specific, absolute-load targets usable without calculation. By leveraging a large-scale dataset of competitive performances, percentile-based normative standards can be derived that reflect the true distribution of strength across body mass categories, providing lift-specific, absolute-load benchmarks directly applicable to athlete assessment. This study aims to provide such a framework, establishing empirical normative percentiles (50th, 75th, 90th, and 99th) for each individual lift and the competitive total, stratified by body mass and sex, as a transparent and data-driven alternative to both informal linear heuristics and aggregate competitive coefficients. The present study focuses exclusively on classic powerlifting: classic lifting has become the primary competitive format within the IPF [5], and the use of supportive equipment introduces additional variability arising from equipment-related factors while limiting the applicability of findings beyond the specific context of equipped competition.

## Methods

### Data Source and Initial Selection Criteria

Data were sourced from the Open Powerlifting database [19]. To ensure a homogeneous population competing under a single, consistent set of technical and anti-doping standards, inclusion was restricted to classic (i.e., unequipped; Equipment = "Raw" in OpenPowerlifting), full-power results (squat, bench press, and deadlift) contested under the IPF and its national affiliates, the international governing body operating a WADA-compliant anti-doping program. Analysis was further limited to competitions held between 1 January 2015 and 8 March 2026, the latter corresponding to the most recent data available at the time of extraction. This contemporary window provides current normative values and limits the blending of performances across eras, consistent with evidence that competitive standards have risen over time [18]. Participant age was restricted to 21–49 years, a broad window designed to safely encompass the observed age of peak competitive performance among sustained raw/classic IPF competitors, reported at approximately 27 years and typically reached around the tenth competition of a lifter's career [20]. Entries with a missing bodyweight or without a successful attempt in each of the three lifts were removed.

### The "Best-Ever" Performance Filter

A critical methodological challenge inherent to large-scale athletic databases is the longitudinal noise introduced when athletes compete multiple times across a career, resulting in significant over-representation of sub-maximal performances. Analyzing every competitive entry for a single athlete would lead to a significant over-representation of sub-maximal efforts and performances, which do not reflect each athlete's peak observed competition result. To resolve this, a "Best-Ever" filter was implemented. Athletes were linked using exact agreement of the Name and Sex fields as supplied by OpenPowerlifting; no additional name-cleaning or quality-control procedures were applied prior to matching. For each unique athlete identified in this way, only the single competition featuring their highest recorded Total was retained for final analysis. Anchoring on the best-total competition, rather than compiling each athlete's best-ever squat, bench press, and deadlift from separate events, preserves the internal coherence of the three lifts as a single, simultaneously achieved performance, avoiding composite results that were never actually realized in competition. Each unique athlete profile therefore contributed one retained observation (*N* = 101,898) (see Additional File – Supplementary Table 1), representing their peak competitive performance at the corresponding body mass.

### Computational Metrics and Performance Tiers

Two primary performance metrics were computed for each athlete within the database. First, the relative strength multiplier was defined as the absolute weight lifted in a specific movement divided by the athlete's recorded body mass at the time of competition. This ratio was employed here as the analytical unit for quantifying the body mass–strength relationship, not as a benchmark in itself: the aim was precisely to test whether the ratio remains constant across body mass, which our results show it does not. The critique advanced in this study is therefore directed at the use of a single, fixed multiplier as a universal standard, not at the relative-strength ratio as a descriptive measure; to ensure the resulting framework does not inherit this limitation, its practical output is expressed as absolute loads in kilograms, requiring no multiplier at the point of use. Second, the internal lift ratio, was defined as the percentage contribution of the squat, bench press, and deadlift to the athlete's competitive total. This dual-metric approach allows for a simultaneous evaluation of an athlete’s strength relative to their mass (external scaling) and their internal distribution across the three lifts (internal scaling) [1, 6, 8]. To provide a comprehensive grading system, the population was segmented into four performance tiers based on statistical distribution: the 50th percentile (Intermediate), 75th percentile (Advanced), 90th percentile (Elite), and the 99th percentile (World Class), following terminology commonly adopted in strength and conditioning practice [21].

### Data Processing and Statistical Analysis

All data were analyzed using R software in RStudio (version 4.3.2). To formally test the hypothesis of mass-dependent decay in relative strength, inferential statistics were applied. Traditional linear regression is heavily influenced by outliers and focuses only on the conditional mean. Therefore, we employed quantile regression [22] to independently estimate and test the specific performance percentiles (50th, 75th, 90th, and 99th). Standard errors and confidence intervals for all quantile regressions were estimated using bootstrap resampling to ensure robust inference and avoid assumption violations, particularly at the extreme quantiles. To assess the robustness of the Best-Ever anchoring strategy for lift-specific values (see Sect. "The "Best-Ever" Performance Filter"), a sensitivity analysis was conducted comparing, for each lift, the percentiles obtained under best-total anchoring against those obtained from each athlete's best-ever single lift, drawn from any qualifying competition irrespective of the corresponding total. To formally assess whether the mass-dependent decline becomes progressively steeper at higher performance tiers, pairwise comparisons of the quantile slopes were conducted using Wald tests for equality of coefficients. Furthermore, model goodness-of-fit across percentiles was evaluated using the Pseudo-R^2^ statistic. This allows for the calculation of statistical significance of the body mass-dependent decline across different tiers of athletic proficiency.

Additionally, linear regression models were used to test for shifting internal lift ratios (the percentage contribution of each lift to the competitive total) as a function of absolute body mass. To provide both theoretical depth and practical utility, the normative analysis employed a dual-modeling approach. First, to evaluate the biological scaling of strength independent of historical administrative boundaries, percentile values were summarized across current IPF weight classes and a Loess (Locally Estimated Scatterplot Smoothing, span = 0.75) procedure was applied to these class-level summaries to visualize mass-dependent trends while reducing categorical noise. Second, to maximize applicability for practitioners, all athletes were retrospectively mapped into current IPF weight classes based on their exact body mass. Because the IPF women's categories were revised within the study window (the 72 kg class replaced by the 69 and 76 kg classes), assignment by recorded body mass, rather than by historical class label, ensures that athletes are classified consistently, regardless of whether they competed before or after this revision. Absolute-load percentile curves (1st–99th) were then generated for each modern category, translating biological trends into directly practical standards for competitive preparation and weight-class transitions.

## Results

### Participant Characteristics and Data Distribution

Following the application of the inclusion criteria and the longitudinal "Best-Ever" filter, the final analysis yielded a robust sample of *N* = 101,898 unique competitive athletes (male: *n* = 67,789; female: *n* = 34,109). This filtered dataset represents the peak drug-tested, classic performance for each individual within the 21- to 49-year age bracket drawn from the International Powerlifting Federation and its affiliates between 1 January 2015 and 8 March 2026.

**Fig. 1:**
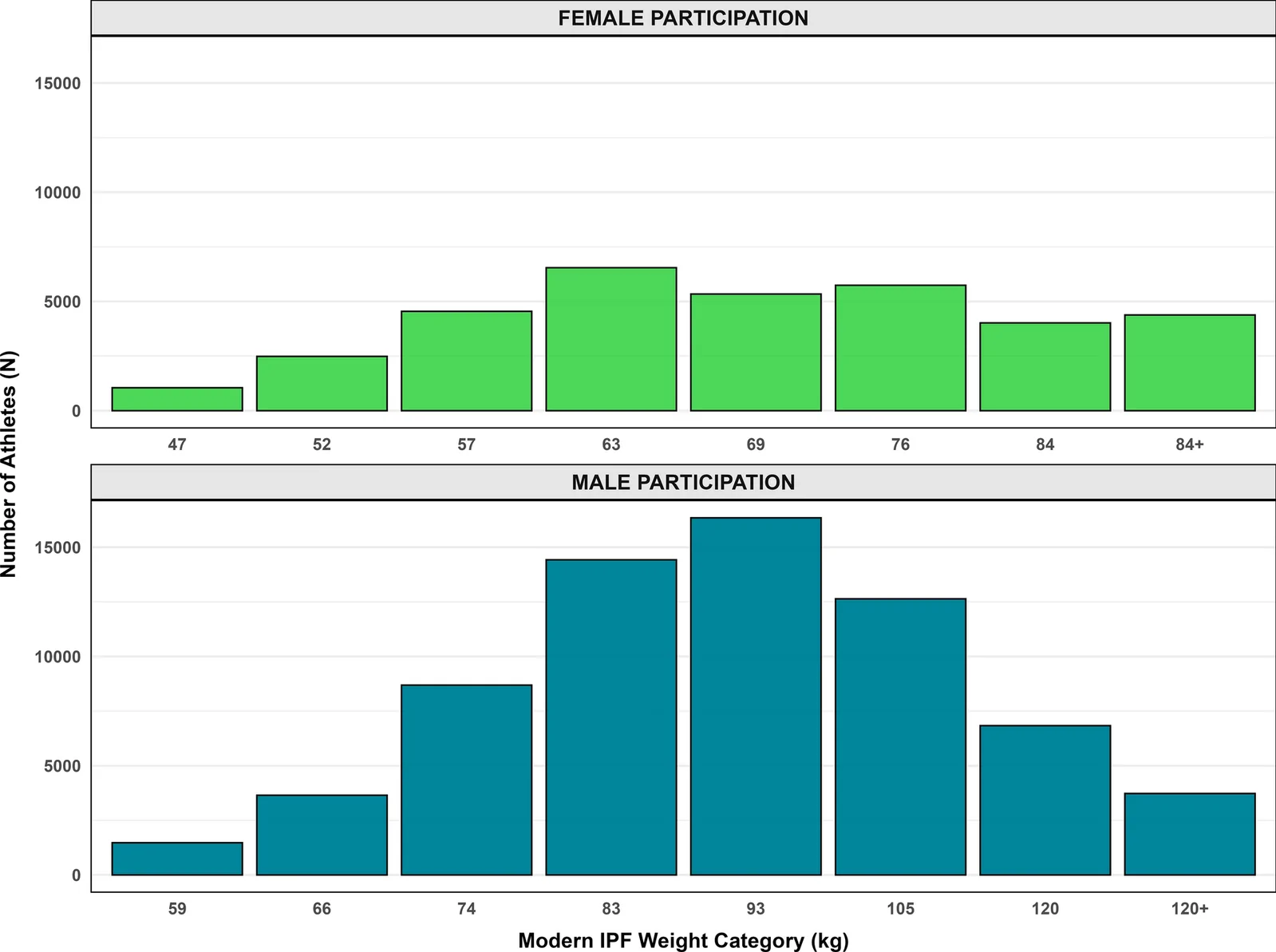
Sample distribution across IPF weight categories. *IPF* International Powerlifting Federation

Descriptive characteristics for each weight category, including medians and interquartile ranges for age, body mass, and competitive total, are detailed in Supplementary Table 2 (see Additional file). The demographic distribution of the sample across the modern IPF weight class structure is illustrated in Fig. 1.

### The Decline of Bodyweight Multipliers

The analysis of BMs revealed a systematic mass-dependent decline across all lifts, performance tiers, and both sexes. Quantile regression analysis showed a highly significant negative mass-dependent decay across all measured percentiles (Table 1).

**Table 1:** Quantile regression outcomes and pairwise slope comparisons

Sex	Lift	Percentile	Beta [SE]	Pseudo-R^2^	Delta vs 50th	*p*-value
Male	Squat	50th (Ref)	−0.0092 [0.0001]	0.078	Reference	–
		75th	−0.0100 [0.0001]	0.086	−0.0008	< 0.001
		90th	−0.0110 [0.0002]	0.094	−0.0018	< 0.001
		99th	−0.0125 [0.0003]	0.112	−0.0033	< 0.001
	Bench press	50th (Ref)	−0.0059 [0.0001]	0.073	Reference	–
		75th	−0.0065 [0.0001]	0.079	−0.0006	< 0.001
		90th	−0.0073 [0.0001]	0.090	−0.0014	< 0.001
		99th	−0.0091 [0.0002]	0.115	−0.0032	< 0.001
	Deadlift	50th (Ref)	−0.0150 [0.0001]	0.179	Reference	–
		75th	−0.0162 [0.0001]	0.189	−0.0013	< 0.001
		90th	−0.0173 [0.0001]	0.195	−0.0023	< 0.001
		99th	−0.0196 [0.0002]	0.205	−0.0047	< 0.001
Female	Squat	50th (Ref)	−0.0111 [0.0001]	0.096	Reference	–
		75th	−0.0117 [0.0002]	0.094	−0.0006	< 0.001
		90th	−0.0125 [0.0003]	0.093	−0.0015	< 0.001
		99th	−0.0136 [0.0005]	0.093	−0.0025	< 0.001
	Bench press	50th (Ref)	−0.0066 [0.0001]	0.102	Reference	–
		75th	−0.0075 [0.0001]	0.095	−0.0009	< 0.001
		90th	−0.0082 [0.0001]	0.093	−0.0016	< 0.001
		99th	−0.0092 [0.0003]	0.089	−0.0026	< 0.001
	Deadlift	50th (Ref)	−0.0161 [0.0001]	0.181	Reference	–
		75th	−0.0175 [0.0002]	0.177	−0.0014	< 0.001
		90th	−0.0188 [0.0002]	0.173	−0.0027	< 0.001
		99th	−0.0192 [0.0006]	0.152	−0.0031	< 0.001

*Beta*: quantile regression slope coefficient representing the absolute reduction in the bodyweight multiplier per 1 kg increase in body mass. *SE*: standard error. *Pseudo-R*^*2*^: goodness-of-fit statistic for quantile regression models. Delta vs 50th: difference in slope coefficient relative to the 50th percentile reference. *p*-value refers to Wald tests for pairwise equality of quantile regression coefficients

Model goodness-of-fit, evaluated via the Pseudo-*R*^2^, ranged from 0.07 to 0.20, with the highest explanatory power consistently observed in the deadlift (Table 1). This indicates that absolute body mass is a statistically significant and practically relevant predictor of the maximum attainable multiplier, specifically in this lift.

The quantile coefficients showed a progressive mass-dependent decline, characterized by a significant steepening of the negative slope from the 50th (Intermediate) to the 99th (World Class) percentile. Pairwise comparisons of the quantile slopes (Wald tests for equality of coefficients) formally confirmed this acceleration, yielding highly significant Δβ parameters across the performance spectrum (Table 1). To clarify the practical magnitude of this mass-dependent decay, the β coefficients reported in Table 1 represent the absolute reduction in the BM for every 1 kg increase in body mass. To put this into perspective, a β of −0.01 indicates that for every 10 kg of additional body mass, an athlete’s expected multiplier drops by exactly 0.1x (e.g., decreasing from a 2.1× to a 2.0× squat multiplier).

**Fig. 2:**
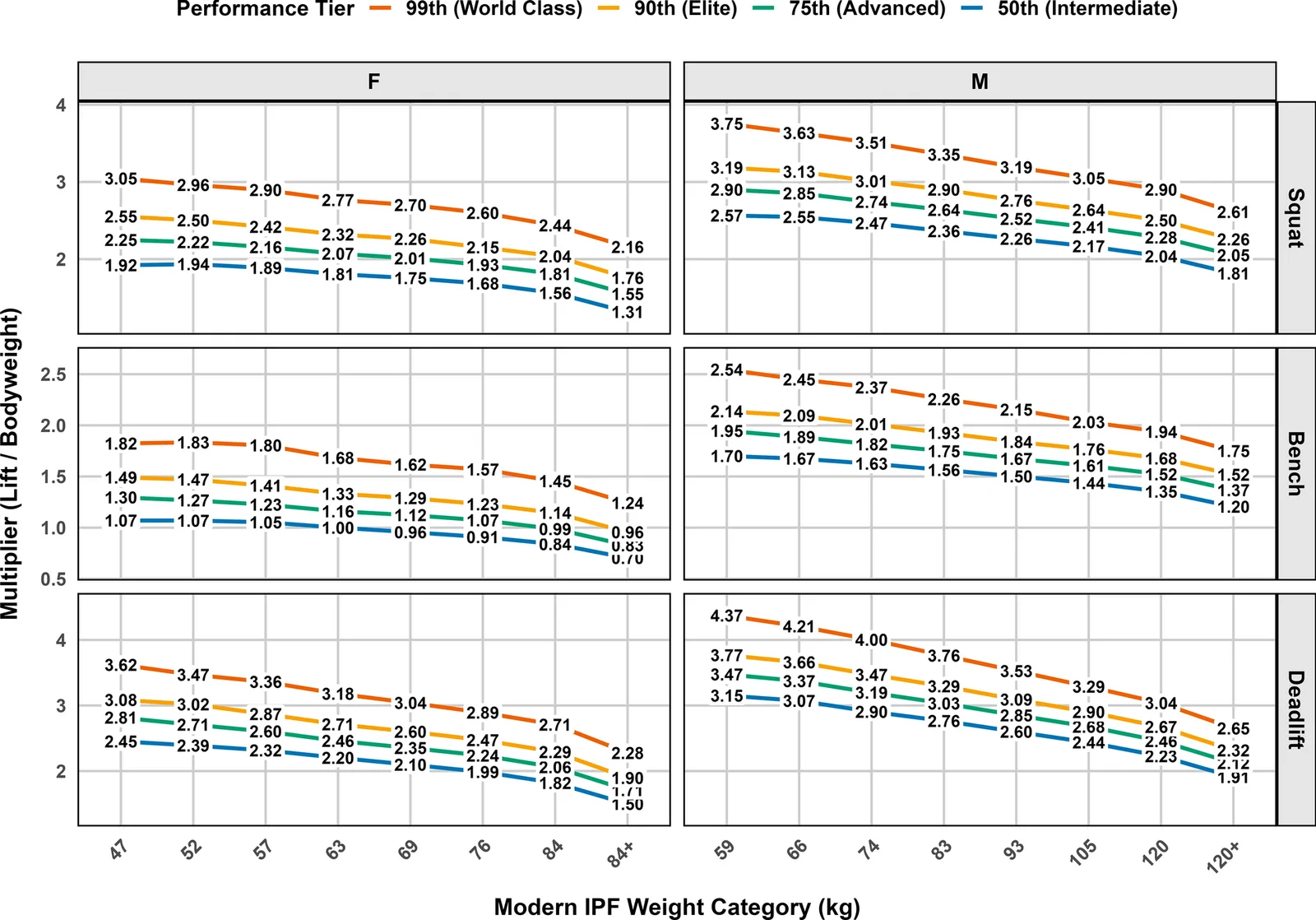
Multiplier decay across all weight categories in the 50th, 75th, 90th and 99th percentiles

Specifically for male athletes, the mass-dependent decay (β) per additional kilogram ranged from −0.0092 to −0.0125 in the squat, from −0.0059 to −0.0091 in the bench press, and from −0.0150 to −0.0196 in the deadlift. A parallel, and slightly steeper, progressive decline was observed in female athletes, with decay coefficients ranging from −0.0111 to −0.0136 in the squat, from −0.0066 to −0.0092 in the bench press, and from −0.0161 to −0.0192 in the deadlift (all *p* < 0.001). Consequently, traditional fixed-ratio benchmarks (e.g., the 2.0× BW squat or 2.5× BW deadlift) become increasingly difficult for heavier athletes to achieve. This systematic erosion of the relative ratio across weight classes is visually summarized in the categorical multiplier matrix (Fig. 2).

To assess whether anchoring on the best-total competition affected these lift-specific multipliers, a sensitivity analysis compared these percentiles against each athlete's best-ever single lift, drawn from any qualifying competition. The two approaches produced near-identical percentiles across all lifts, sexes, and performance tiers, with a maximum difference of ≤ 0.02 × body mass (Additional file – Supplementary Table 3). This difference was conservative in direction, as best-total anchoring can only under-estimate, never inflate, the reported single-lift norms, confirming that the Best-Ever filter did not meaningfully bias the lift-specific normative values.

**Fig. 3:**
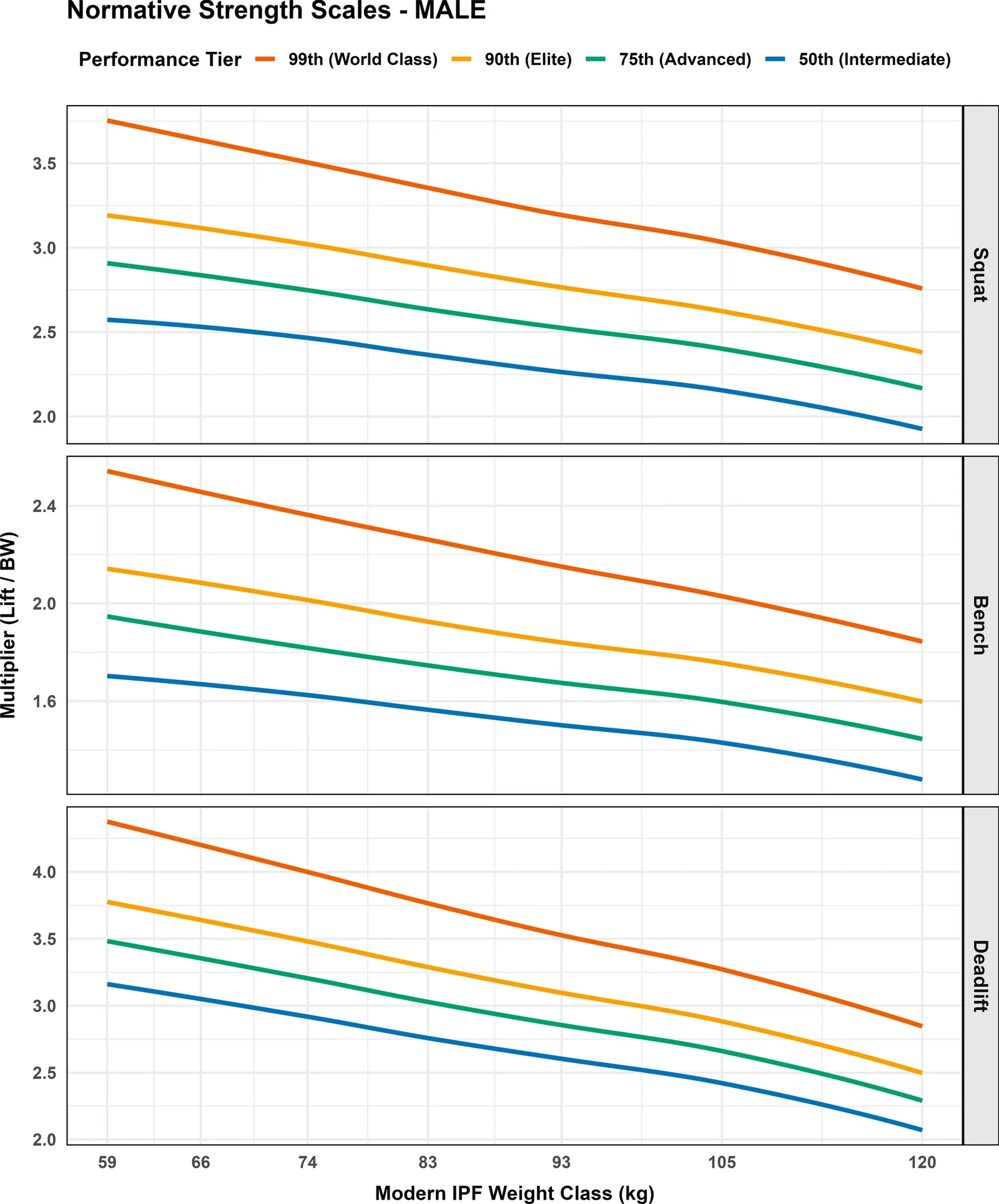
Male normative strength scales. Lines represent Loess-smoothed decrease in bodyweight multipliers across weight classes, shown for four percentiles of the squat, bench, and deadlift. *Loess* Locally Estimated Scatterplot Smoothing

**Fig. 4:**
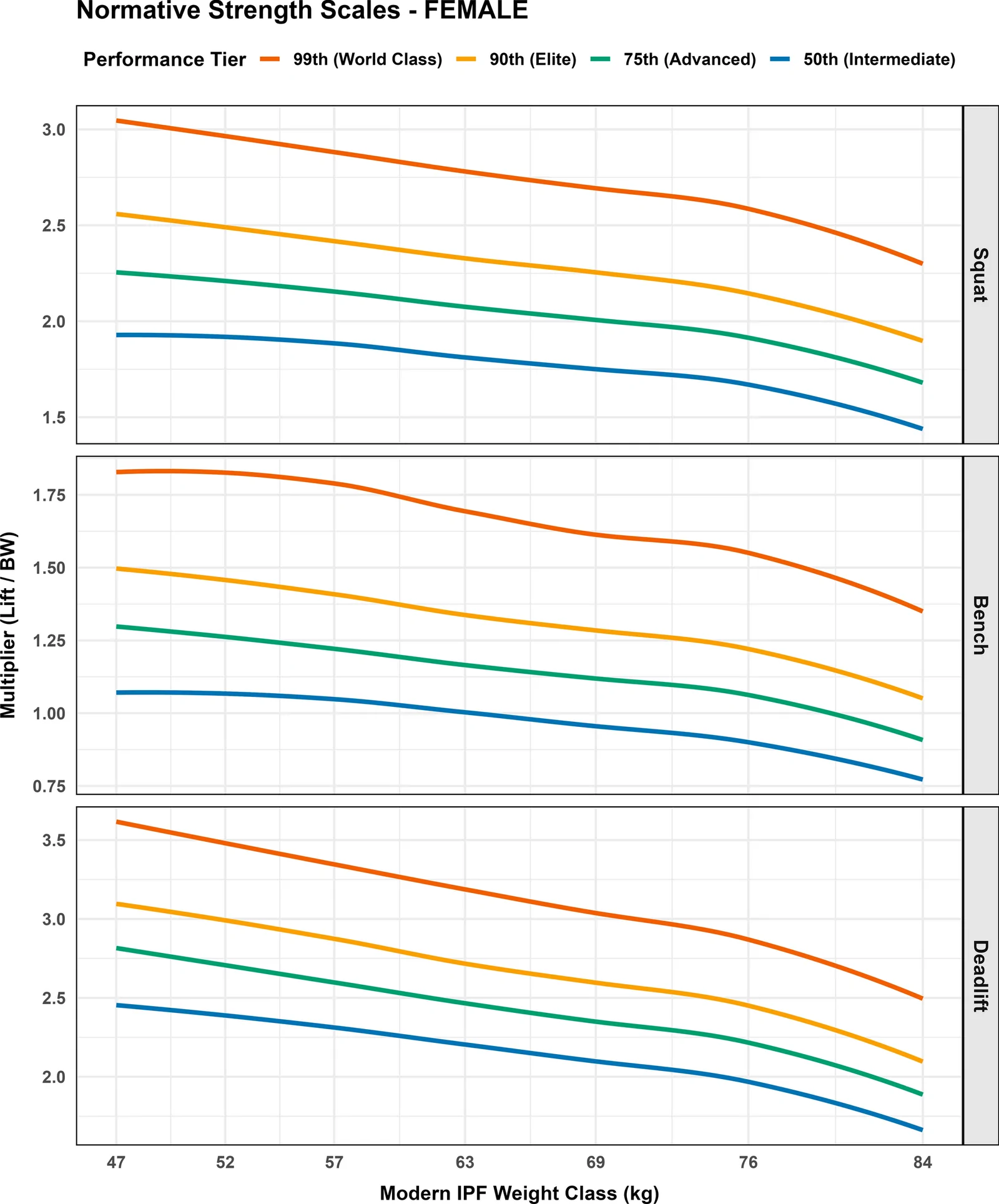
Female normative strength scales. Lines represent Loess-smoothed decrease in bodyweight multipliers across weight classes, shown for four percentiles of the squat, bench, and deadlift. *Loess* Locally Estimated Scatterplot Smoothing

To translate these relative constraints into comprehensive biological and practical models, percentile values were evaluated across the modern weight-class spectrum. The output of the Loess smoothing procedure applied to these class-level summaries resulted in distinct, non-intersecting performance lanes for each lift (Figs. 3 and 4).

These smoothed curves provide a visually continuous representation of mass-dependent decay across weight classes, though the underlying quantile regressions are linear models and do not formally test for nonlinearity. The strict non-intersection of these curves confirms the structural validity of the model, indicating that the algorithm accurately preserved the expected hierarchy of performance tiers (i.e., 99th > 90th > 75th > 50th percentiles) across weight classes, consistent with the mass-dependent decay shown in the quantile regressions.

**Fig. 5:**
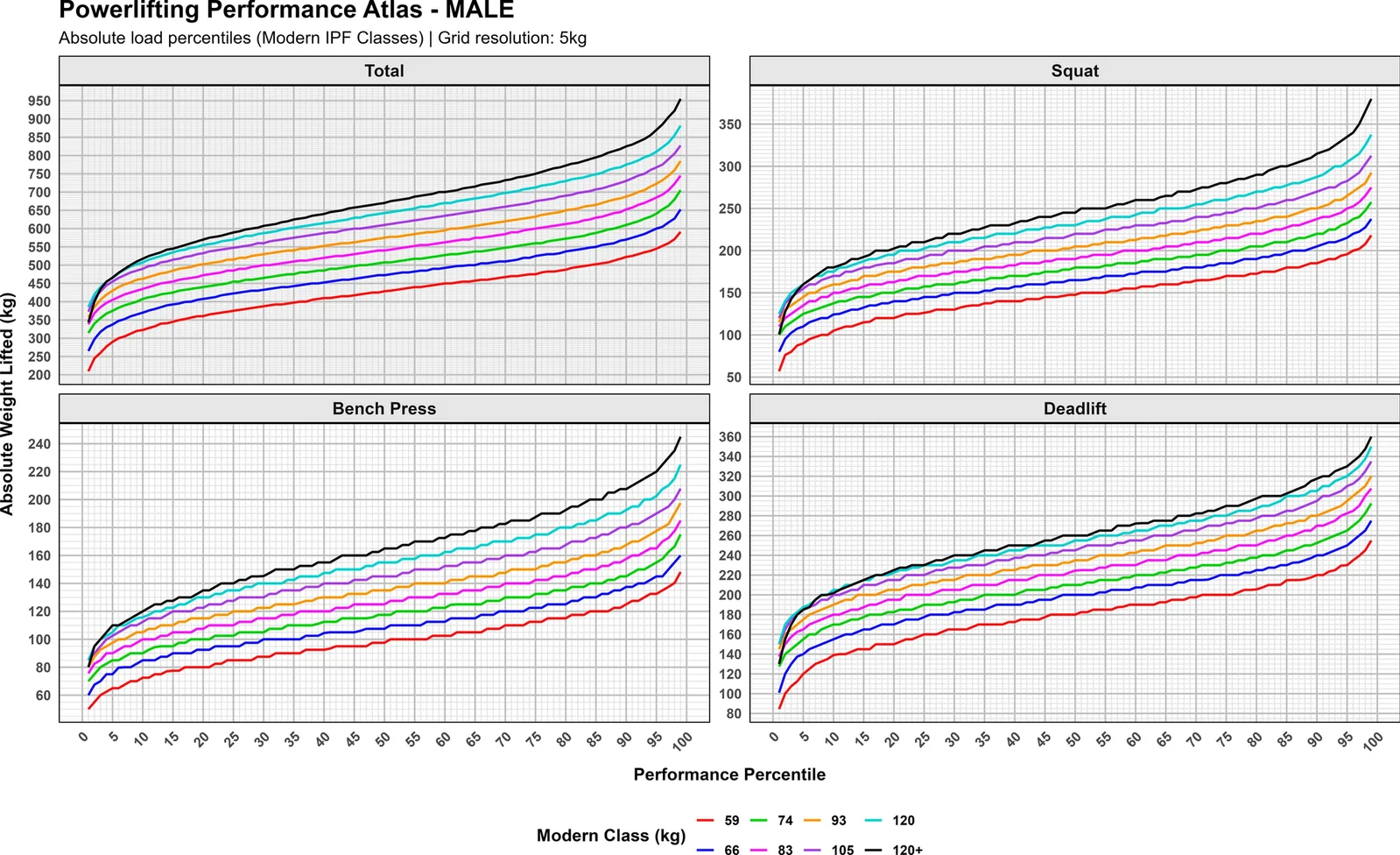
Male powerlifting performance atlas

**Fig. 6:**
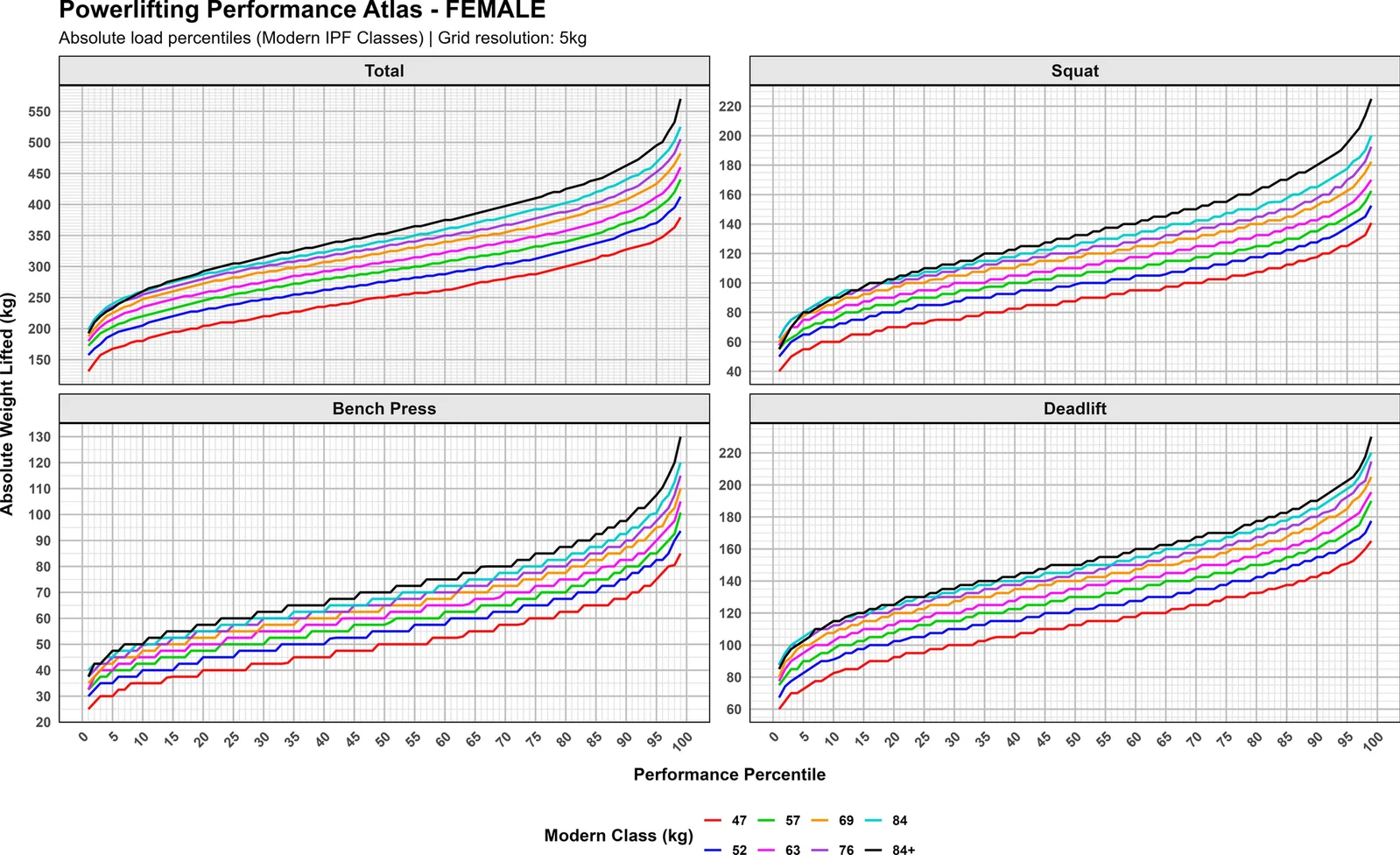
Female powerlifting performance atlas

Building upon this theoretical framework to maximize field applicability, the empirical data were subsequently transformed into absolute-load (kg) normative curves specific to modern IPF categories. These absolute normative quad-panels (Figs. 5 and 6) provide a definitive, data-driven alternative to the linear approach traditionally used in the strength and conditioning community, allowing coaches to benchmark athletes in raw kilograms while intrinsically accounting for the mass-dependent decline paradigm. Finally, individual, high-resolution normative atlases for each lift and sex have been provided as a standalone, printable toolkit in Additional File (Supplementary Figures S1-S8).

### Mass-Dependent Shift in the Internal Lift Ratio

**Fig. 7:**
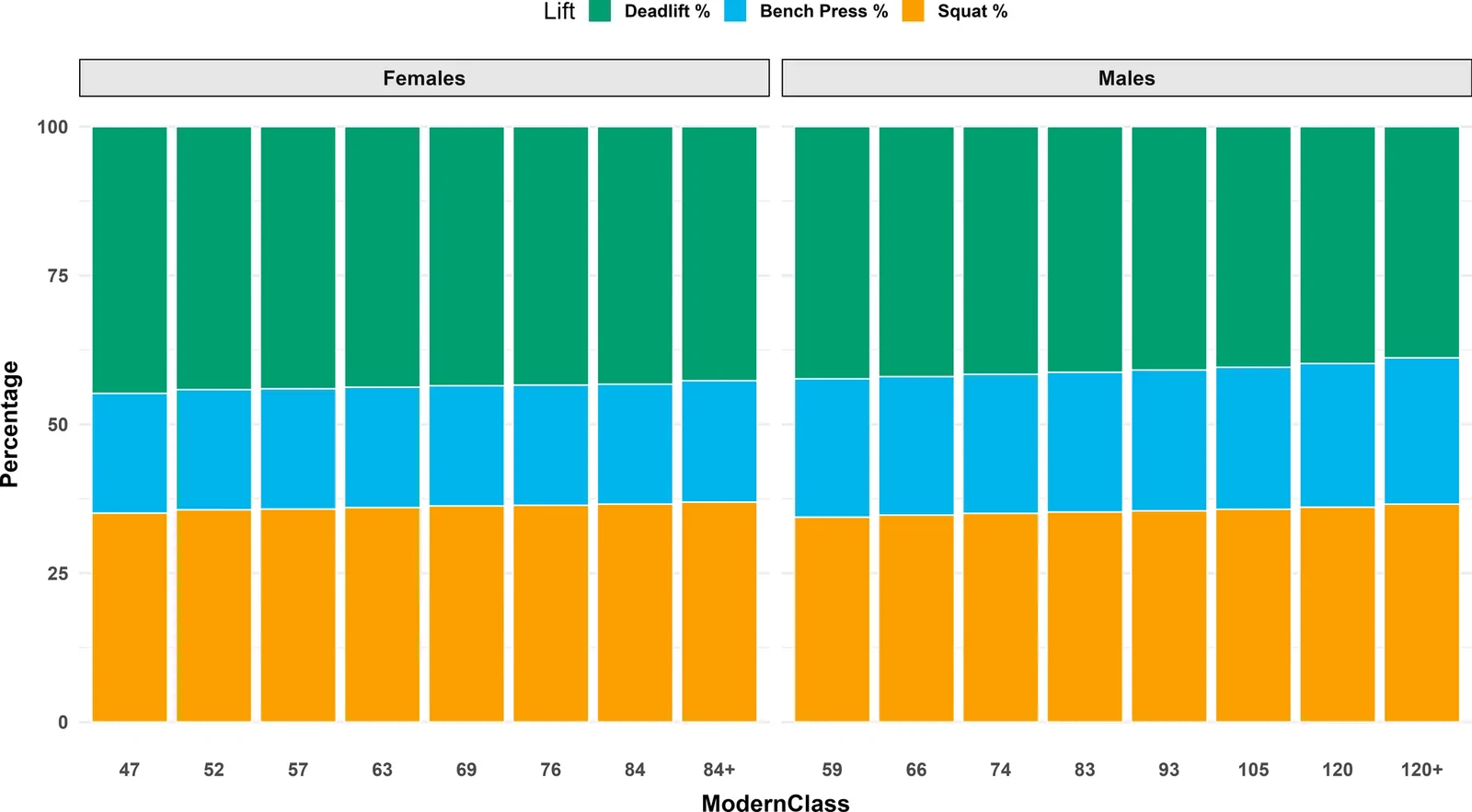
Internal lift ratio distribution by sex and weight class. Relative contribution of individual lifts (squat, bench, deadlift) to the competitive total for females (left) and males (right)

Beyond the decrease in absolute multipliers, the analysis identified a systematic shift in the internal distribution of strength, termed the "internal lift ratio" (Fig. 7).

In the lightweight categories, the deadlift represents the dominant contributor to the competitive total, accounting for approximately 42–45% of the total weight lifted. However, as body mass increases, this dominance undergoes a systematic decline, with the deadlift's contribution shrinking as the squat's contribution expands, narrowing the gap between the two lifts substantially in super-heavyweight categories (e.g., from an 8-percentage-point difference in the lightest male class to approximately 2 percentage points in the heaviest).

Linear regression models indicated a statistically significant association between body mass and these shifts (Table 2). The modest *R*^2^ values reflect the univariate nature of the models and the expected residual variance attributable to individual morphological and technical factors; the inferential objective of these models was to characterize the directional shift in lift contribution as a function of body mass, not to construct a predictive framework. For both sexes, absolute body mass was a significant negative predictor of the deadlift's percentage contribution. In male athletes, this reduction was offset by a proportional increase in both the squat and the bench press, whereas in female athletes, the decline in deadlift importance was almost exclusively compensated for by an increase in the squat ratio (Table 2). Notably, the bench press proportion in females remained virtually static across the entire mass spectrum, as evidenced by a negligible effect size. Because the squat, bench press, and deadlift percentages sum to 100% by definition, these three trends are not fully independent: a decline in one lift's contribution is partly algebraically compensated by an increase in the others, and this dependence should be considered when interpreting the individual regression models in Table 2.

**Table 2:** Results of linear regression models for shifting internal lift ratios as a function of body mass

Sex	Lift	b (Estimate)	95% CI	F-statistic (df)	*p*-value	*R*^2^
Male	Squat	+0.0266	[+ 0.0257, +0.0276]	F(1, 67787) = 3034.2	< 0.001	0.0428
	Bench Press	+0.0176	[+ 0.0166, +0.0187]	F(1, 67787) = 1065.0	< 0.001	0.0155
	Deadlift	−0.0442	[−0.0453, −0.0432]	F(1, 67787) = 6817.9	< 0.001	0.0914
Female	Squat	+0.0266	[+ 0.0249, +0.0284]	F(1, 34107) = 875.6	< 0.001	0.0250
	Bench press	+0.0051	[+ 0.0035, +0.0067]	F(1, 34107) = 39.4	< 0.001	0.0012
	Deadlift	−0.0318	[−0.0337, −0.0299]	F(1, 34107) = 1073.9	< 0.001	0.0305

*β*: regression slope coefficient representing the change in lift percentage contribution per 1 kg increase in absolute body mass. *95% CI* 95% confidence interval. *F-statistic*: result of the *F-test* for the linear regression model with degrees of freedom in parentheses. *R*^*2*^: coefficient of determination

## Discussion

This study aimed to establish empirical, percentile-based normative standards for strength assessment in competitive powerlifting, providing a practitioner-oriented alternative to traditional linear bodyweight multipliers. The findings confirm that static BM benchmarks are not equally attainable across the full body-mass spectrum: the capacity to express relative strength is not a static trait but undergoes a systematic decline as absolute body mass increases. That maximal strength declines with increasing body mass is, in itself, well established and follows directly from geometric scaling principles. A theoretical baseline for this pattern is provided by established principles of biological scaling and the structural constraints of human physiology [6], which we adopt here as an interpretive framework rather than as a mechanism formally tested in the present analysis. The primary driver of this decline is the non-linear relationship between muscle cross-sectional area and total body volume, often described by the Square-Cube Law [7]. As an athlete scales upwards in size, their mass increases according to the cube of their linear dimensions (L3), while their strength-generating capacity, which is functionally dependent on the cross-sectional area of the contractile tissue, increases only by the square (L2) [7]. This mathematical constraint disrupts the assumption of geometric isometry: a heavier athlete will inherently possess a lower strength-to-mass ratio than a lighter counterpart of equivalent training status.

A novel and highly relevant finding of this investigation is the progressive nature of the mass-dependent decline revealed by the quantile regression coefficients. Our contribution lies not in the existence of a mass-dependent decline [6, 7, 17], but in demonstrating that its rate is not uniform: using quantile regression, we show that the slope of this decline steepens systematically across performance tiers, a structure not predicted by a single allometric exponent and, to our knowledge, not previously quantified. While the Square-Cube Law provides a theoretical baseline for strength decay, our data indicate that the slope of this decay (β) becomes significantly steeper as performance level increases, reflecting significant heterogeneity across performance tiers. Rather than a uniform decline, the slope parameters steepen as athletes approach the upper percentiles of the distribution, indicating that the mass-dependent decline in relative strength is not constant but scales with athletic proficiency [16, 23]. The non-overlapping confidence intervals between intermediate and world-class cohorts further suggest a diminishing efficiency of scale. This is likely driven by differences in the composition of weight gain across training ages rather than an increased physiological cost of new muscle. Muscle hypertrophy follows a linear-log trajectory, with training-related strength gains reaching a practical plateau over time [24]; advanced lifters may therefore experience diminishing returns in muscle accretion relative to less trained individuals. Consequently, when elite lifters gain weight, a higher proportion of that mass may be non-contractile tissue compared to intermediates. Despite this, contractile tissue remains the primary driver of force, as muscle mass strongly predicts performance even among elite powerlifters [25].

For coaches and high-performance practitioners, these data provide an additional, descriptive data point to inform weight-class strategy; such decisions should nonetheless remain primarily guided by health and physiological considerations, including avoidance of conditions such as Relative Energy Deficiency in Sport, for which the atlas provides no information. At the 99th percentile, the observed decline in relative strength is steeper, meaning that even a modest increase in body mass corresponds to a comparatively larger reduction in the relative multiplier; a speculative interpretation is that this may reflect a lower margin for non-functional mass gain at this tier, though body composition was not assessed in the present analysis. Consequently, the transition to a higher weight category for a world-class athlete may require a larger increase in absolute performance to preserve relative standing compared to the same transition for an intermediate lifter, an observation that can inform, but should not replace, individualized, health-first category decisions.

The observed decrease in our results often exceeds the theoretical predictions of isometric scaling, suggesting that secondary physiological factors worsen the loss of relative strength in heavier weight classes. One contributing factor is the theoretical ceiling imposed by the fat-free mass index (FFMI) [26]. As athletes approach the upper boundaries of their natural hypertrophic potential, further increases in total body mass may be disproportionately composed of non-contractile tissues, such as skeletal density, connective tissue, and adipose mass [16, 23]. However, carrying higher fat mass may also concomitantly permit a higher total FFMI, as observed in sumo wrestlers [27], by allowing greater total lean mass gains despite the lower proportion of total mass being contractile tissue, also evidenced by the fact that leaner individuals lose a higher proportion of lean mass when attempting to reduce body weight [28]. This non-functional tissue nonetheless increases the total mass that must be displaced without contributing to the force-generating numerator, thereby inflating the denominator of the BM ratio. This offers one plausible explanation for why, in the world-class 99th percentile tier, the rate of relative strength loss is more pronounced in the super-heavyweight categories, where the pursuit of absolute total often comes at the expense of lean body mass percentage.

The mass-dependent shift in the internal lift ratio of the three powerlifts is particularly important. The transition from a markedly deadlift-dominant profile in lightweights to a substantially narrower margin between deadlift and squat contribution in heavyweights represents a systematic mass-dependent shift to increased body volume. This describes a population-level trend rather than a rule holding for every individual: substantial variation exists around this pattern, and some athletes deviate considerably from it. This structural shift was validated by linear modeling, which identified body mass as a highly significant negative predictor of the deadlift's percentage contribution and a significant positive predictor of the squat's contribution (Table 2). The non-overlapping, narrow confidence intervals confirm the precision of these estimates: each 10 kg increase in body mass is associated with a ~0.44 percentage point reduction in the deadlift's share of the total and a ~0.28 percentage point increase in the squat's contribution, a meaningful, systematic shift that compounds substantially across the full body mass spectrum. This finding provides a basis for hypothesizing that increased body volume alters mechanical leverages, potentially penalizing the deadlift at the starting position, the lockout, or both, while potentially facilitating the squat through increased joint stability and reduced effective range of motion [14, 16].

This shift may be driven by the phenomenon of soft tissue interference [14]. In the deadlift, excessive abdominal and thigh girth contribution can compromise the starting position, forcing the athlete to adopt a wider stance or a more rounded spinal profile, which increases the distance between the barbell and the hip joint's center of mass [15]. This mechanical disadvantage increases the torque requirements for the posterior chain, potentially limiting the deadlift’s scaling potential [15]. An equally plausible mechanism operates at the lockout, where increased abdominal and thigh girth may impede the bar's path past the thighs during final hip and knee extension; our data cannot distinguish which of these two mechanisms, or their combination, better accounts for the observed pattern. In contrast, the squat may benefit from increased body volume through mechanisms that remain to be empirically confirmed, including greater passive joint stability and a potential reduction in effective range of motion through soft tissue compression at the bottom of the movement [14, 16].

The identified discrepancy in upper-body scaling further emphasizes the need for sex-specific normative standards. The steeper decrease in female bench press performance compared to the posterior chain suggests the female upper body is subject to a steeper mass-dependent decline as mass increases. While this may be attributed to biological differences in regional muscle distribution and activation [29], it may also be influenced by systematic, sociological factors, such as differences in prior exposure to upper-body dominant training or occupational tasks between sexes. Supporting this, Roberts et al. [30] demonstrated that females make significantly larger relative gains in upper-body strength than males in response to training (with hypertrophy and lower-body gains being similar between sexes), possibly reflecting a lower initial upper-body training age. Thus, the observed discrepancy could reflect a combination of physiological and environmental/sociological factors. Our quantile models further highlight a slightly steeper decay in the female squat compared to males (e.g., β = −0.0136 vs β = −0.0125 at the 99th percentile). In practical terms, this means that for every 10 kg increase in body mass, a world-class female athlete is expected to lose 0.136 xBW in relative squat strength compared to 0.125 xBW in her male counterpart, a difference of 0.011 xBW per 10 kg that compounds progressively across weight categories. This emphasizes that female athletes exhibit a steeper mass-dependent decline in these lifts as they move into heavier weight classes. Consequently, applying male-derived ratios or multipliers to female athletes is not only descriptively inaccurate but sets targets that do not correspond to the observed female distribution. The proposed normative framework accounts for this by providing independent, data-driven sex-specific benchmarks.

From a practical coaching perspective, the transition from a linear approach to a percentile-based normative system allows for a more informative and body mass-adjusted assessment of athletic progress. By comparing an athlete's individual lifts against the observed distribution of competitive powerlifters at the same body mass and sex, these normative standards allow practitioners to distinguish results that fall within the expected mass-dependent range from those that diverge from it. The interpretation of such divergence, whether it reflects a modifiable training gap, an anthropometric or morphological specialization, or an injury-related constraint, remains the coach's or clinician's responsibility, informed by contextual information the atlas does not contain. Furthermore, plotting an athlete's trajectory across the 50th, 75th, 90th, and 99th percentiles provides a framework for long-term development. Critically, stratification by IPF weight class, as detailed in Additional File (see Supplementary Figures S1-S8), enables direct cross-category comparison, providing an empirical basis for evidence-based decisions regarding weight class transitions. To maximize field applicability, these normative standards have been translated into absolute-load (kg) atlases stratified by IPF weight class, sex, and individual lift, provided as a printable toolkit in Additional File (see Supplementary Figures S1-S8). This allows coaches to benchmark athletes directly in kilograms lifted, without requiring any calculation.

While this study utilizes one of the largest datasets in S&C research specifically related to powerlifters, several limitations need to be mentioned. First, although restricting the sample to a contemporary window (2015–2026) limits era-blending, normative values in this population remain subject to secular drift: recent evidence indicates that squat and deadlift medians can rise by approximately 2.5–7.5 kg within five-year periods [18]. The atlases presented here should therefore be regarded as current reference values rather than fixed benchmarks, and we recommend periodic re-derivation (e.g., every five years) to maintain their contemporary accuracy. Second, because the Best-Ever filter selects each athlete's highest recorded competitive result, athletes with more qualifying competitions have more opportunities to record a higher value, an expected property of any peak-selection design rather than a correctable bias. We consider this preferable to including every qualifying entry, which would conflate an athlete's peak with their average performance and understate the observed distribution of competitive results. Additionally, because this filter anchors on the highest-total competition, an athlete's peak in an individual lift may occasionally have occurred at a different event; a sensitivity analysis confirmed this had a negligible, conservative effect on the lift-specific percentiles (≤ 0.02 × body mass; Additional file—Supplementary Table 3). A related limitation concerns athlete identification itself: athletes were identified using the name and sex fields supplied by OpenPowerlifting, and variations in spelling, punctuation, transliteration, or name changes may cause results from the same athlete to be assigned to separate database profiles. Consequently, although one observation was retained per unique database profile, it cannot be excluded that a small number of athletes contributed more than one retained observation; no probabilistic record-linkage procedure was undertaken. Third, the body mass values recorded in the database represent the athletes' recorded mass at the time of the official weigh-in, not necessarily their actual body mass during the lift. Powerlifters frequently engage in short-term body mass manipulation [31], reducing body mass ahead of the weigh-in and subsequently refeeding and rehydrating before competing, so that they step onto the platform at a higher body mass than their official weight class limit. This may result in an overestimation of bodyweight multipliers, particularly in lighter and middleweight categories where this practice is highly prevalent [31]. Furthermore, Kwan and Helms [32] observed that medal-winning powerlifters at the world championships manipulated body mass to a greater extent than non-medalists. Consequently, the discrepancy between weigh-in mass and actual platform mass could be larger in our 99th percentile cohort compared to our 50th percentile cohort, which may further confound the comparative rate of multiplier decay. Fourth, the retrospective nature of the database precludes the analysis of individual anthropometric variables, such as limb-length and torso-to-leg ratio, which are known to influence lift-specific performance [16] and remain an uncontrolled confound in this analysis. Future research should extend this framework to equipped and untested powerlifting divisions, and to other strength sports where weight-class stratification is relevant.

## Practical Applications

This research provides a data-driven framework that coaches and practitioners can implement directly in their assessment and programming workflows. Rather than relying on single linear benchmarks or diagnostic percentile-lookup tools that report only where an athlete currently stands, coaches should locate an athlete within their respective IPF weight class using the normative atlases provided in Additional File (see Supplementary Figures S1-S8) and identify their percentile tier across each lift individually in absolute kilograms, making any lift-specific asymmetry against the reference distribution directly visible. An athlete may present at the 75th percentile in the deadlift while remaining at the 50th in the bench press, a discrepancy that a total-based linear multiplier would obscure entirely. The interpretation of such a divergence, whether it reflects a modifiable training gap or an individual morphological specialization (e.g., anthropometric limb-length proportions that inherently favor one lift over another) remains the coach's responsibility, informed by contextual knowledge the atlas does not capture. The atlas is not intended to replace established training-programming and periodization practices, which remain appropriately based on the athlete's current strength and training status. Its role is narrower: when an athlete or coach is weighing whether, and by how much, to move up a weight category, the atlas provides the absolute-load target associated with a given percentile in the destination class, a longer-term reference point rather than a substitute for programming. Given the progressive mass-dependent decline documented in this study, this matters because a fixed relative benchmark does not carry over across weight categories: an athlete moving into a heavier category should expect their relative BM to decrease even with maintained absolute strength, and the atlas allows the specific kilogram target in the new category to be identified directly. Finally, practitioners working with female athletes should apply the sex-specific normative tables exclusively, rather than adapting male-derived benchmarks. The steeper mass-dependent decline observed in female bench press and squat performance is not captured by male standards. Applying male BM to female athletes does not merely introduce descriptive inaccuracy: it sets targets that do not correspond to the observed female distribution, with direct consequences for athlete motivation and long-term development. The normative atlases provided in Additional File (see Supplementary Figures S1-S8) offer a direct, calculation-free reference tool for all of the above applications, allowing coaches to benchmark athletes and set lift-specific targets in raw kilograms for each IPF weight class and sex, without requiring any further calculation.

## Conclusion

This study demonstrated that the traditional use of a single linear BM as a universal standard is not equally attainable across body-mass classes and fails to account for the non-linear scaling of strength with body mass. We have identified a systematic decrease in relative strength and a systematic shift in the proportional contribution of individual lifts to the competitive total as body mass increases. By establishing a new empirical normative framework based on the actual performance distribution of over 101,000 competitive athletes, and by translating these findings into absolute-load normative atlases stratified by IPF weight class and sex, we provide a robust alternative to both simplistic heuristics and existing competitive coefficients. These findings confirm that percentile-based standards represent a more empirically calibrated framework for strength assessment compared to traditional linear heuristics.

Practically, this framework enables coaches and practitioners to set lift-specific targets in absolute kilograms that account for the mass-dependent scaling of human performance, replacing arbitrary informal heuristics with a transparent, evidence-based tool applicable across all weight categories and both sexes.

## Supplementary Information

Additional file 1.Supplementary Table 1 (sample flow through inclusion criteria and the "Best-Ever" filter); Supplementary Table 2 (descriptive characteristics for each weight category, including medians and interquartile ranges for age, body mass, and competitive total); Supplementary Table 3 (sensitivity analysis comparing lift-specific percentiles under best-total versus best-lift anchoring, by sex, lift, and performance tier); Supplementary Figures S1-S8 (individual, high-resolution normative atlases for each lift and sex, provided as a standalone printable toolkit)

## Data Availability

Publicly available datasets were analyzed in this study, sourced from the Open Powerlifting project database (https://www.openpowerlifting.org/). The exact dataset extract used for the analyses (accessed 8 March 2026), the R code used for data processing, filtering, and statistical analysis, and high-resolution versions of the normative atlases (Supplementary Figures S1-S8) suitable for printing are publicly available at the Open Science Framework (OSF): [https://doi.org/10.17605/OSF.IO/P4HZG].

## Abbreviations

BM: Bodyweight multiplier
BMs: Bodyweight multipliers
BW: Bodyweight
S&C: Strength and conditioning
IPF: International Powerlifting Federation
Loess: Locally Estimated Scatterplot Smoothing
FFMI: Fat-free mass index
CI: Confidence interval
SE: Standard error
xBW: Times bodyweight (e.g., 0.133 xBW)
GL: IPF GL formula (Good Lift)
