# Supplementary material for: Percentile-Based Normative Standards for Strength Assessment Beyond Linear Bodyweight Multipliers in Competitive Powerlifters
Source: Sports Med Open. 2026 Sep 16;12:136. doi: 10.1186/s40798-026-01111-z (PMC13582683; doi:10.1186/s40798-026-01111-z)
Supplement: Supplementary file 1 [file 40798_2026_1111_MOESM1_ESM.docx]

Sports Medicine - Open

**Percentile-Based Normative Standards for Strength Assessment Beyond Linear Bodyweight Multipliers in Competitive Powerlifters**

Simone Montenegro¹,², Pamela Wicker³*, Tim Wiedenmann¹, Ludwig Rappelt¹, Lars Donath¹

¹Department of Applied Exercise Science, German Sport University Cologne, Cologne, Germany
²Department of Medicine and Aging Sciences, Università degli Studi "G. d'Annunzio" Chieti-Pescara, Chieti, Italy
³Department of Sports Science, Bielefeld University, Bielefeld, Germany

*Correspondence: Pamela Wicker; [pamela.wicker@uni-bielefeld.de](mailto:pamela.wicker@uni-bielefeld.de)

Supplementary Table 1. Sample flow through inclusion criteria and the "Best-Ever" filter.

| Step | N remaining | N excluded |
| --- | --- | --- |
| Initial records (raw export) | 3,868,570 | 0 |
| Event = SBD (full power) | 2,800,587 | 1,067,983 |
| Equipment = Raw (classic) | 1,100,483 | 1,700,104 |
| ParentFederation = IPF | 578,900 | 521,583 |
| Tested = Yes | 578,582 | 318 |
| Date within 2015-01-01 to 2026-03-08 | 531,232 | 47,350 |
| Successful attempt in all three lifts | 512,612 | 18,620 |
| Age 21-49 years | 264,561 | 248,051 |
| Non-missing bodyweight and valid sex | 264,510 | 51 |
| Valid weight class | 262,411 | 2,099 |
| Best-Ever filter (one observation per athlete profile) | 101,898 | 160,513 |
| N remaining: number of records retained after the given step. N excluded: number of records removed at that step relative to the preceding step. SBD: squat-bench-deadlift (full-power) event. IPF: International Powerlifting Federation. | | |

Supplementary Table 2. Participant characteristics across current IPF weight categories.

| Sex | WeightClassKg | N | Age (yrs) | Body Mass (kg) | Total (kg) |
| --- | --- | --- | --- | --- | --- |
| F | 47 | 1,050 | 25.5 [22.5 - 31.5] | 46.3 [45.5 - 46.7] | 250.0 [210.0 - 287.5] |
|  | 52 | 2,485 | 26.5 [23.0 - 32.5] | 51.2 [50.5 - 51.6] | 275.0 [239.0 - 312.5] |
|  | 57 | 4,549 | 26.5 [23.0 - 32.0] | 56.0 [55.3 - 56.5] | 292.5 [255.0 - 332.5] |
|  | 63 | 6,545 | 27.0 [23.5 - 32.5] | 61.7 [60.6 - 62.4] | 307.5 [267.5 - 347.5] |
|  | 69 | 5,340 | 27.0 [23.5 - 32.5] | 67.3 [66.0 - 68.2] | 322.5 [282.5 - 365.0] |
|  | 76 | 5,740 | 27.5 [24.0 - 33.5] | 72.3 [70.8 - 74.5] | 332.5 [290.0 - 377.5] |
|  | 84 | 4,019 | 28.5 [24.5 - 35.5] | 81.4 [79.2 - 82.8] | 340.0 [297.5 - 392.5] |
|  | 84+ | 4,381 | 31.5 [26.0 - 38.5] | 98.5 [91.2 - 109.7] | 352.5 [305.0 - 410.0] |
| M | 59 | 1,475 | 25.0 [22.5 - 30.5] | 58.3 [57.4 - 58.7] | 428.6 [375.0 - 475.0] |
|  | 66 | 3,649 | 25.0 [22.5 - 30.0] | 65.2 [64.4 - 65.6] | 472.5 [422.5 - 525.0] |
|  | 74 | 8,690 | 25.5 [22.5 - 29.5] | 72.9 [71.9 - 73.5] | 507.5 [455.0 - 560.0] |
|  | 83 | 14,426 | 25.5 [22.5 - 30.5] | 81.5 [80.1 - 82.2] | 540.0 [485.0 - 600.0] |
|  | 93 | 16,347 | 26.5 [23.0 - 31.5] | 91.0 [89.0 - 92.0] | 575.0 [515.0 - 632.6] |
|  | 105 | 12,640 | 27.0 [23.5 - 32.5] | 102.0 [99.0 - 103.6] | 610.0 [547.5 - 675.0] |
|  | 120 | 6,834 | 28.5 [24.5 - 34.5] | 114.9 [110.8 - 117.9] | 642.5 [570.0 - 712.5] |
|  | 120+ | 3,728 | 30.0 [25.0 - 36.5] | 134.1 [126.8 - 145.3] | 670.0 [589.4 - 750.0] |

Values are presented as median [interquartile range, 25th–75th percentile], except for N (count). WeightClassKg: current IPF weight category, in kilograms. Age: age at competition, in years. Body Mass: recorded body mass at weigh-in, in kilograms. Total: sum of the best successful squat, bench press, and deadlift attempts, in kilograms. IPF: International Powerlifting Federation.

Supplementary Table 3. Sensitivity analysis comparing lift-specific percentiles under best-total versus best-lift anchoring.

| **Sex** | **Lift** | **Anchoring** | **50th** | **75th** | **90th** | **99th** |
| --- | --- | --- | --- | --- | --- | --- |
| Male | Squat | Best-Total (main analysis) | 2.26 | 2.55 | 2.83 | 3.34 |
|  |  | Best-Lift (sensitivity) | 2.26 | 2.56 | 2.84 | 3.35 |
| Female |  | Best-Total (main analysis) | 1.71 | 2.00 | 2.27 | 2.76 |
|  |  | Best-Lift (sensitivity) | 1.71 | 2.00 | 2.27 | 2.77 |
| Male | Bench Press | Best-Total (main analysis) | 1.50 | 1.69 | 1.88 | 2.25 |
|  |  | Best-Lift (sensitivity) | 1.50 | 1.70 | 1.89 | 2.26 |
| Female |  | Best-Total (main analysis) | 0.93 | 1.11 | 1.30 | 1.67 |
|  |  | Best-Lift (sensitivity) | 0.94 | 1.12 | 1.31 | 1.68 |
| Male | Deadlift | Best-Total (main analysis) | 2.59 | 2.92 | 3.23 | 3.81 |
|  |  | Best-Lift (sensitivity) | 2.61 | 2.94 | 3.25 | 3.84 |
| Female |  | Best-Total (main analysis) | 2.04 | 2.37 | 2.67 | 3.22 |
|  |  | Best-Lift (sensitivity) | 2.05 | 2.38 | 2.69 | 3.23 |
| Best-Total (main analysis): lift-specific multiplier taken from each athlete's highest-Total competition. Best-Lift (sensitivity): lift-specific multiplier taken from each athlete's best-ever performance in that lift, drawn from any qualifying competition. Values expressed as multiples of body mass (x BW). Maximum absolute difference between anchoring strategies across all lifts, sexes, and percentiles: 0.02 x BW. | | | | | | |

***Supplementary Figure S1.*** Female powerlifting performance atlas: Bench Press

***Supplementary Figure S2.*** Female powerlifting performance atlas: Deadlift

***Supplementary Figure S3.*** Female powerlifting performance atlas: Squat

***Supplementary Figure S4.*** Female powerlifting performance atlas: Total

***Supplementary Figure S5.*** Male powerlifting performance atlas: Bench press

***Supplementary Figure S6.*** Male powerlifting performance atlas: Deadlift

***Supplementary Figure S7.*** Male powerlifting performance atlas: Squat

***Supplementary Figure S8.*** Male powerlifting performance atlas: Total
